# Long noncoding RNA SOX2OT promotes pancreatic cancer cell migration and invasion through destabilizing FUS protein via ubiquitination

**DOI:** 10.1038/s41420-021-00640-8

**Published:** 2021-09-22

**Authors:** Yan Wang, Xiong-Fei Zhang, Dong-Yan Wang, Yi Zhu, Lei Chen, Jing-Jing Zhang

**Affiliations:** 1 Endoscopy Center, The First Affiliated Hospital of Nanjing Medical University, Nanjing, 210029 People’s Republic of China; 2 Department of Biochemistry, Nanjing University of Chinese Medicine, Nanjing, 210023 People’s Republic of China; 3 Department of General Surgery, Affiliated Hospital of Integrated Traditional Chinese and Western Medicine, Nanjing University of Chinese Medicine, Nanjing, 210028 Jiangsu People’s Republic of China; 4 Department of General Surgery, The First Affiliated Hospital of Nanjing Medical University, Nanjing, 210029 People’s Republic of China; 5 Pancreas Center, The First Affiliated Hospital of Nanjing Medical University, Nanjing, 210029 People’s Republic of China; 6 Pancreas Institute of Nanjing Medical University, Nanjing, 210029 People’s Republic of China

**Keywords:** Oncogenes, Pancreatic cancer

## Abstract

Pancreatic cancer is a highly aggressive and lethal digestive system malignancy. Our previous studies revealed the correlation of high levels of lncRNA SOX2OT expression with patients’ poor survival outcomes, the promoting role of SOX2OT in proliferation and cycle progression of pancreatic cancer cells, and the in vivo binding of SOX2OT to RNA binding protein FUS, which destabilized the protein expression of FUS. However, the mechanism of SOX2OT binding and inhibiting FUS protein stability remains unclear. In this study, we performed RNA pull-down, cycloheximide-chase, and ubiquitination assays to determine the effect of SOX2OT on FUS ubiquitination, and explored the specific regulatory mechanism of SOX2OT–FUS axis in pancreatic cancer cell migration, invasion, in vivo tumor growth, and metastasis through RNA sequencing. We found that SOX2OT binds to FUS through its 5′ and 3′ regions, resulting in FUS ubiquitination and degradation. The SOX2OT–FUS regulatory axis promotes migration, invasion, tumor growth, and metastasis ability of pancreatic cancer cells. The in-depth elaboration of the SOX2OT–FUS regulatory axis in pancreatic cancer may clarify the mechanism of action of SOX2OT and provide new ideas for pancreatic cancer treatment.

## Introduction

Pancreatic cancer is a highly aggressive and lethal digestive system malignancy. According to the latest report, pancreatic cancer is currently the fourth leading cause of cancer-related death in the world and the sixth in China. Due to the limited means of diagnosis and treatment of pancreatic cancer, its 5-year survival rate is only 8% [1, 2]. Therefore, it is urgent to explore effective therapeutic targets for pancreatic cancer, which depends on the in-depth exploration of the pathogenesis and development mechanism of pancreatic cancer.

With the completion of the human genome project, it has been found that the number of protein-coding genes in the human genome is less than 30,000, accounting for only 2% of the whole-genome sequence, while the remaining 98% of the genome sequences produce a large number of noncoding RNA (ncRNA), which constitute a complex RNA regulatory network in cells. Among them, long ncRNA (lncRNA) is a kind of RNA with a length of more than 200 nt and does not have the ability to encode protein. LncRNA accounts for about 90% of the total genome transcripts, far more than the proportion of protein-coding RNA [3]. LncRNA often contains conserved local regions in its molecular structure, and its expression is spatiotemporal specific. In recent years, a large number of evidences have confirmed that lncRNA can be used as a broad-spectrum regulatory factor, playing an important regulatory role in all aspects of gene expression, such as chromosome modification, transcription, and posttranscription [4]. LncRNA is closely related to the occurrence and development of tumor, and participates in the proliferation, apoptosis, and invasion of tumor cells and distant metastasis [5–7]. Although many achievements have been made in the research of lncRNA in recent years, the function of a large part of lncRNA is still unclear.

In our previous study, we found that the expression of lncRNA SOX2OT was upregulated in pancreatic cancer, and it was associated with poor prognosis of pancreatic cancer patients [8]. In vivo and in vitro functional assays confirmed that SOX2OT promoted the proliferation, clone formation, and cycle progression of pancreatic ductal adenocarcinoma cells [9]. With the chromatin isolation by RNA purification (ChIRP) and RNA binding protein immunoprecipitation (RIP) assays that were performed in previous study, we found that SOX2OT directly binds to FUS in vivo, an hnRNP protein that contains one RNA recognition motif, and reduced the stability of FUS, without changing the FUS mRNA expression [9].

FUS (also known as TLS) is a multifunctional RNA binding protein, which is mainly located in the nucleus and related to multiple steps of RNA metabolism, including transcription, splicing, microRNA (miRNA) processing, mRNA transport, and local translation [10, 11]. Sato et al. [12] demonstrated the interaction between β-catenin and FUS/TLS or other RNA binding proteins involved in regulating the splicing process of mRNA precursors. Brooke et al. [13] also confirmed that FUS is a key molecule connecting androgen receptor signaling pathway and cell cycle progression in prostate cancer. Studies have shown that FUS is abnormally expressed in some tumors, such as liposarcoma, breast cancer, cervical cancer, prostate cancer, and liver cancer, and is related to the malignant progression of tumors [14–16].

However, the mechanism of SOX2OT binding and inhibiting FUS protein stability remains unclear, and the role of SOX2OT–FUS regulatory axis in pancreatic cancer cell migration and invasion is still unclear. To further explore the mechanism through which SOX2OT modulates the stability of FUS protein, we performed RNA pull-down, cycloheximide (CHX)-chase, and ubiquitination assays to determine the regulatory effect of SOX2OT on FUS ubiquitination. We also explored the specific regulatory mechanism of SOX2OT and its downstream protein FUS in pancreatic cancer cell migration and invasion.

## Results

### Effect of SOX2OT–FUS regulatory axis on the in vivo tumor formation and metastasis ability of pancreatic cancer cells

**Fig. 1:**
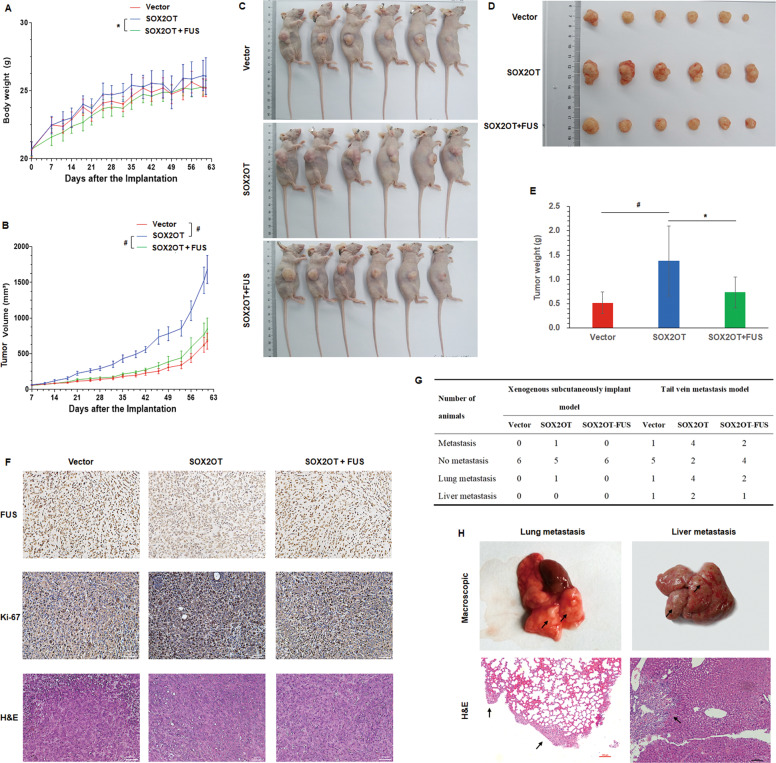
SOX2OT–FUS regulatory axis promotes in vivo tumor formation and metastasis ability of pancreatic cancer cells. Body weight diversity curves (**A**) and tumor growth curves (**B**) in mice over time after subcutaneous injection with 1 × 10^6^ of the indicated cells. In vivo (**C**) and in vitro (**D**) photos of tumors at the end of the experiment in subcutaneous tumorigenesis models. **E** Tumor weight of the subcutaneous tumors taken from mice. **F** HE staining and immunohistochemistry analysis of FUS and Ki67 in subcutaneous tumors. Magnification, ×100. **G** Metastasis in subcutaneous tumorigenesis models and tail vein metastasis models. **H** In tail vein metastasis models, lung and liver metastases were evaluated by macroscopic observation and by histomorphology under microscopy. Scale bar, 100 μm. The arrows indicate the metastases. (* represents *p* < 0.05, # represents *p* < 0.001, compared with the control group).

To further study the effects of SOX2OT–FUS regulatory axis on tumorigenicity and metastasis of pancreatic cancer, in vivo experiments were performed via the subcutaneous or tail vein injection of transduced cells (from the PANC-1–Vector, PANC-1–SOX2OT, and PANC-1–SOX2OT–FUS group) into BALB/c-nude mice. After injection, in the xenograft tumor model, the results showed that the body weight (Fig. 1A), tumor volume (Fig. 1B–D), and tumor weight (Fig. 1E) were larger and the tumor formation rate was faster in SOX2OT group than in vector group. SOX2OT–FUS group had smaller subcutaneous tumors and slower rate of tumor formation than SOX2OT group. The difference was statistically significant (*p* < 0.05). In addition, cell viability was also increased in the tumors of SOX2OT overexpression cells as determined by Ki67 staining (Fig. 1F). These results indicated that SOX2OT also promotes pancreatic cancer cell growth in vivo.

In the tail vein metastasis model, lung or liver metastases were observed in only one of the six mice injected with PANC-1–Vector group. By contrast, metastases were observed in four of the six mice injected with PANC-1–SOX2OT group. However, metastases were observed in only two of the six mice injected with PANC-1–SOX2OT–FUS group (Fig. 1G, H). In addition, we also observed the metastasis in the xenograft models. No metastasis was found in PANC-1–Vector and PANC-1–SOX2OT–FUS group, whereas there were one lung metastases in PANC-1–SOX2OT group (Fig. 1G, H). However, there was no significant difference among the three groups. It may be because the sample size is too small, but the trend of the results showed that overexpression of SOX2OT promotes pancreatic cancer cell metastasis in vivo.

These results revealed that SOX2OT had positive regulatory effects on the tumor growth and metastasis of pancreatic cancer cells and FUS overexpression could obviously reverse the carcinogenesis caused by SOX2OT in PANC-1 cell lines.

### Effect of SOX2OT–FUS regulatory axis on the in vitro migration and invasion ability of pancreatic cancer cells

**Fig. 2:**
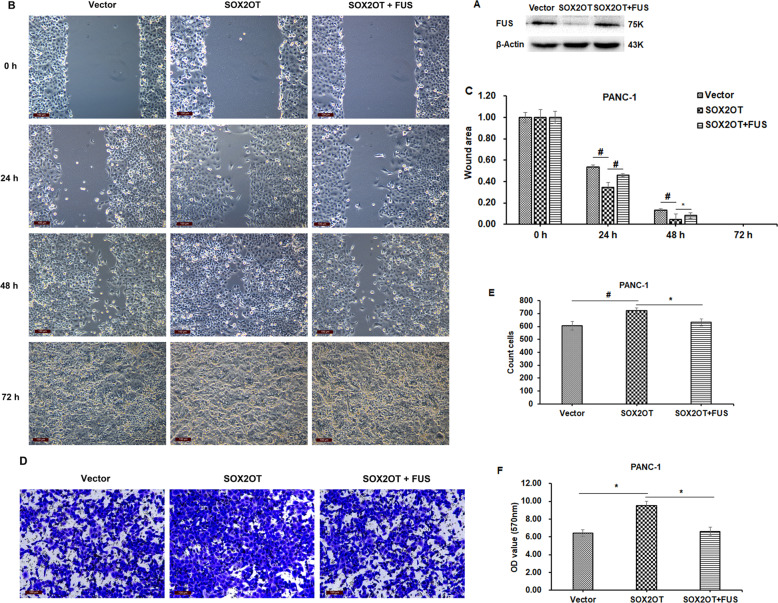
SOX2OT–FUS regulatory axis promotes in vitro migration and invasion ability of pancreatic cancer cells. **A** FUS expression in PANC-1–Vector, PANC-1–SOX2OT, and PANC-1–SOX2OT–FUS cells was confirmed by western blot analysis. **B** Cell wound healing assays were performed. Phase images were taken at 0, 24, 48, and 72 h, respectively. Magnification, ×100. The relative wound areas (**C**) were measured by Image-Pro Plus software. **D** Cellular transwell assays were performed. The membranes of the chambers were stained with 0.1% crystal violet and photographed. Magnification, ×200. **E** The cells in five random fields of view were counted. **F** The stained cells were soaked in 33% acetic acid and the absorbance of 33% acetic acid containing crystal violet was assessed using a microplate reader at a 570 nm wavelength. The optical density (OD) value indirectly reflected the number of penetrated cells. (* represents *p* < 0.05, # represents *p* < 0.001, compared with the control group).

We investigated the effect of SOX2OT–FUS regulatory axis on cell migration with cell wound healing assay. The results in Fig. 2B, C showed that the wound healing rate of SOX2OT-overexpressing cells was significantly faster than that of the control group, while FUS overexpression could obviously reverse the effect caused by SOX2OT in PANC-1 cell lines. These results revealed that FUS had negative regulatory effects on the migration of pancreatic cancer cells and could obviously reverse the migration promoting role caused by SOX2OT in the PANC-1 cell lines.

We also investigated the effect of SOX2OT–FUS regulatory axis on cell invasion with cellular transwell assay. As shown in Fig. 2D–F, SOX2OT overexpression significantly promoted the invasion of PANC-1 cells compared with the control group, while FUS overexpression could obviously reverse the effect caused by SOX2OT in PANC-1 cell lines. These results revealed that FUS had negative regulatory effects on the invasion of pancreatic cancer cells and could obviously reverse the invasion promoting role caused by SOX2OT in the PANC-1 cell lines.

### SOX2OT binds directly to FUS protein through its 1–887 nt and 2663–3550 nt sequence

**Fig. 3:**
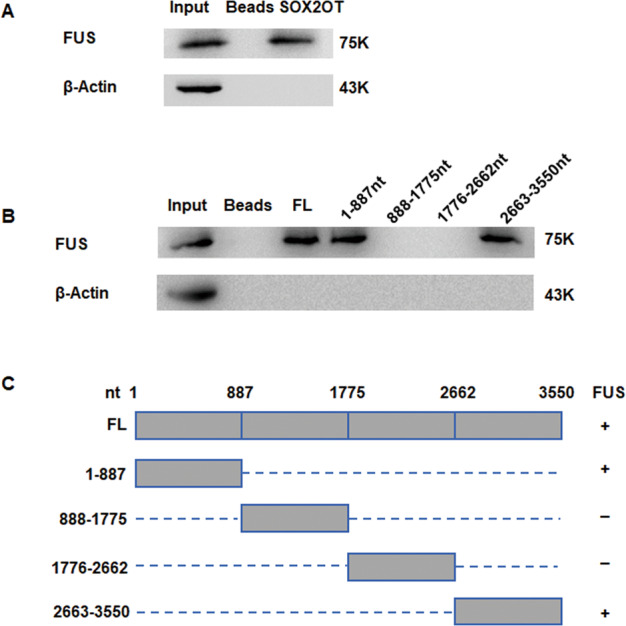
SOX2OT binds directly to FUS protein through its 1–887 nt and 2663–3550 nt sequence. **A** RNA pull-down assay followed by western blot confirmed FUS as a protein partner binding specifically to SOX2OT. β-Actin is protein controls. **B** Western blot of FUS pulled-down by truncated SOX2OT fragments. This analysis revealed that nucleotides 1–887 and 2663–3550 of SOX2OT (SOX2OT 1–887 nt, SOX2OT 2663–3550 nt) are sufficient to interact with FUS protein, while other SOX2OT truncated fragments could not. FL full length. **C** Schematic of truncated or deletion mutants of SOX2OT. The FUS binding capability is shown (right).

Previously, we have verified the direct in vivo binding relationship between SOX2OT and FUS through ChIRP and RIP experiments [9]. In this study, the bands specific to SOX2OT were obtained in vitro by RNA pull-down assay and subjected to western blot analysis for protein identification. As shown in Fig. 3A, FUS protein band appeared in SOX2OT group and input group, but not in beads only group. These results indicated that SOX2OT could directly bind to FUS protein in vitro.

To map the SOX2OT functional motifs corresponding to FUS binding, we conducted an in vitro RNA pull-down assay using a series of truncated SOX2OT fragments (Fig. 3C). This analysis revealed that nucleotides 1–887 and 2663–3550 of SOX2OT (SOX2OT 1–887 nt, SOX2OT 2663–3550 nt) are sufficient to interact with FUS protein, while other SOX2OT truncated fragments could not (Fig. 3B). These data suggested that the SOX2OT 5′ (1–887 nt) and 3′ (2663–3550 nt) regions are necessary for SOX2OT’S binding to FUS protein. We will further design more truncated or deletion mutants of the SOX2OT 1–887 nt and 2663–3550 nt regions to map with greater precision the sequence of SOX2OT that binds to FUS.

### SOX2OT promotes the ubiquitination and degradation of FUS protein

In our previous study, we measured FUS expression at the transcriptional level and protein level in the SOX2OT overexpression cell lines, we found that SOX2OT affected FUS protein level, but not mRNA level [9]. Thus, we could reasonably hypothesize that the instability of FUS was caused by the binding of SOX2OT and FUS.

**Fig. 4:**
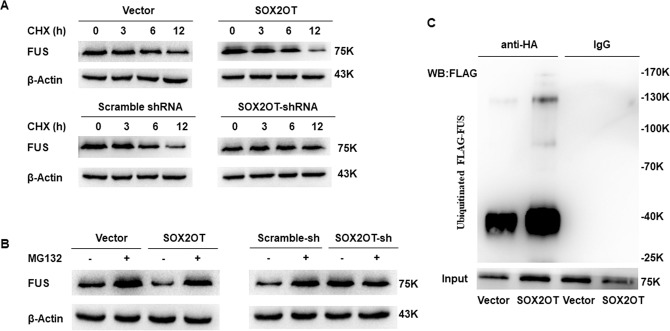
SOX2OT promotes the ubiquitination and degradation of FUS protein. **A** Cells were treated with the protein synthesis inhibitor CHX, and the remaining of FUS protein was measured by western blot at 0, 3, 6, and 12 h. **B** Western blot analysis of FUS in the SOX2OT overexpression or SOX2OT knockdown cells treated with MG132 (+) or DMSO (–) for 6 h. **C** The SOX2OT overexpression cells were cotransfected with a plasmid expressing a HA-tagged ubiquitin (HA-Ub) and a plasmid expressing a FLAG-tagged FUS (FLAG-FUS). After MG132 treatment, cell lysates were prepared and subjected to IP using an anti-HA antibody. The ubiquitinated FLAG-FUS was further detected by western blot using an anti-FLAG antibody.

The cells from SOX2OT, Vector, SOX2OT shRNA, and Scramble shRNA groups were treated with the protein synthesis inhibitor CHX, and the expression level of FUS protein was determined at 0, 3, 6, and 12 h. The results showed that the degradation rate of FUS protein was significantly higher in SOX2OT group than in the vector group. The degradation rate of FUS protein significantly decreased in SOX2OT shRNA group as compared with the Scramble shRNA group (Fig. 4A). These results indicated that SOX2OT could inhibit the stability of FUS protein.

The cells from SOX2OT, Vector, SOX2OT shRNA, and Scramble shRNA groups were treated with the proteasome inhibitor MG132, to block the degradation of proteins through proteasome. The difference in FUS protein expression was determined by western blot analysis. MG132 treatment resulted in the accumulation of FUS in SOX2OT overexpression or SOX2OT knockdown cells to the level comparable to that of the control cells (Fig. 4B), which demonstrated that FUS is a proteasome substrate, and the relative higher level of FUS in untreated SOX2OT knockdown cells is largely due to the reduction of FUS degradation.

We then measured the ubiquitination of FUS in the SOX2OT overexpression cells cotransfected with a plasmid expressing a HA-tagged ubiquitin (HA-Ub) and a plasmid expressing a FLAG-tagged FUS (FLAG-FUS). After MG132 treatment, the ubiquitinated FLAG- FUS protein was captured by HA immunoprecipitation (IP) and detected by western blot using an anti-FLAG antibody. The extent of FUS ubiquitination was increased markedly in the SOX2OT overexpression cells (Fig. 4C), which suggests a positive regulatory role of SOX2OT in the process of FUS ubiquitination.

### The downstream target genes and signaling pathways regulated by SOX2OT–FUS axis

**Fig. 5:**
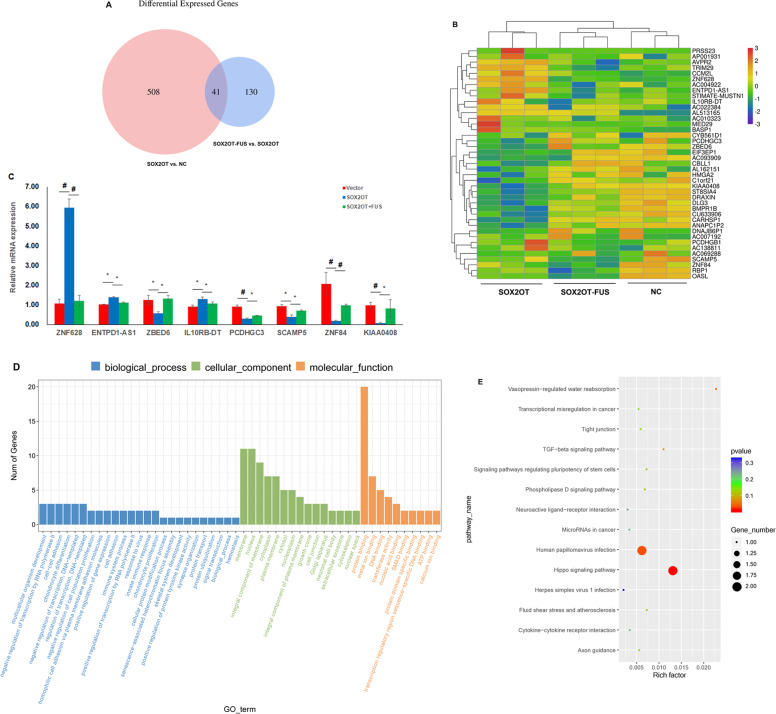
The downstream target genes and signaling pathways regulated by SOX2OT–FUS axis. **A** Venn diagram showing that the overlapped genes between the differentially expressed genes of PANC-1–SOX2OT & PANC-1–Vector and PANC-1–SOX2OT–FUS & PANC-1–SOX2OT by RNA-seq. **B** A heatmap showing 41 differentially expressed genes may be regulated by SOX2OT–FUS axis. **C** qRT-PCR validation of the expression changes of several SOX2OT–FUS axis regulated genes that have been implicated in cancer. * represents *p* < 0.05, # represents *p* < 0.001, compared with the control group. **D** The histogram of Gene Ontology (GO) enrichment analysis results reflects the number distribution of genes with significant differences in GO term enriched by biological process, cellular component, and molecular function. **E** ggplot2 is used to display the KEGG pathway enrichment analysis results in a scatter diagram. Rich factor indicates the number of differential genes located in the KEGG/the total number of genes located in the KEGG. The greater the rich factor value, the greater the KEGG enrichment degree. In the scatter diagram, the size of the point represents S gene number, the color of the point represents the *p* value of enrichment analysis, that is, the significance of enrichment, and *p* value less than or equal to 0.05 represents significant enrichment.

RNA sequencing (RNA-seq) was performed to identify potential SOX2OT–FUS axis target genes and signaling pathways in PANC-1 cells. The results showed that there were 549 genes which were differentially expressed between PANC-1–SOX2OT group and PANC-1–Vector group (Additional file 1, Table S1). Of the 549 genes, 207 were upregulated and 342 were downregulated in SOX2OT overexpression PANC-1 cells. And there were 171 genes that were differentially expressed between PANC-1–SOX2OT group and PANC-1–SOX2OT–FUS group (Additional file 2, Table S2). Of the 171 genes, 54 were upregulated and 117 were downregulated in FUS overexpression PANC-1–SOX2OT cells. The intersection analysis was performed between the differentially expressed genes of PANC-1–SOX2OT & PANC-1–Vector and PANC-1–SOX2OT–FUS & PANC-1–SOX2OT (Additional file 3, Table S3). There were 41 genes regulated by SOX2OT, which can be reversed by overexpression of FUS, which means that these genes may be regulated by SOX2OT–FUS axis (Fig. 5A, B). Among these genes, we selected eight genes (ZBED6, PCDHGC3, SCAMP5, ZNF628, ZNF84, ENTPD1-AS1, IL10RB-DT, and KIAA0408) with obvious differences and verified them by quantitative RT-PCR (qRT-PCR). The qRT-PCR data for these genes were consistent with those obtained by RNA-seq (Fig. 5C).

Gene Ontology (GO) functional enrichment analysis for these 41 differentially expressed genes revealed that the SOX2OT–FUS regulated genes are mainly enriched for the molecular function and cellular component. Most genes that corresponded to cellular component were involved in membrane and nucleus. Protein binding and DNA binding were the most prevalent in molecular function (Fig. 5D). KEGG pathway enrichment analysis revealed that the SOX2OT–FUS regulated genes are mainly enriched for the pathways of Hippo signaling pathway, TGF-beta signaling pathway, and pathways in cancer (Fig. 5E).

## Discussion

In recent years, lncRNA SOX2OT has been proved to play a special role in many diseases. More and more studies have shown that SOX2OT can affect different types of tumors. For example, SOX2OT can downregulate the expression of SOX3 by regulating miR-194-5p and miR-122, so as to regulate JAK/STAT pathway, inhibit the transcriptional expression of TDGF-1, and affect the biological behavior of glioblastoma stem cells [17]. Shahryari et al. [18] also reported the coregulation of SOX2OT and its new splicing variants SOX2OT-s1 and SOX2OT-s2 with key stem cell pluripotency genes SOX2 and OCT4, which are involved in the development of esophageal squamous cell carcinoma. Wang et al. [19] showed that epigallocatechin gallate targeting SOX2OT transcripts has synergistic effect with adriamycin to inhibit the growth of osteosarcoma cells. In 2018, Li et al. [20] found that exosomal lncRNA SOX2OT, derived from a highly invasive pancreatic ductal adenocarcinoma, promotes the invasion and metastasis of pancreatic ductal adenocarcinoma by acting as a competitive endogenous RNA (ceRNA) to induce EMT and stem cell-like characteristics. However, the expression pattern and mechanism of SOX2OT in pancreatic ductal adenocarcinoma have not been fully elucidated.

The mechanisms of role of lncRNA can be summarized into four modes [21–24]. (1) As a signal transduction molecule, lncRNA participates in the transmission of special signal pathways. (2) LncRNA as the bait to bind transcription factor or transcription regulator, thereby blocking the role and signal pathway of the molecule, or bind miRNA as ceRNA, thereby preventing miRNA from binding to its target mRNA. (3) Similar to chaperone molecules, lncRNA binds to proteins (usually transcription factors), which can locate protein complexes on specific DNA sequences to regulate the transcription of downstream molecules. (4) lncRNA can also play the role of central platform, that is, multiple related transcription factors can be bound to this lncRNA molecule, so as to achieve noninterference. In addition, it was also found that some lncRNAs can directly bind to proteins and affect the posttranslational modification of proteins, such as phosphorylation, ubiquitination, acetylation, glycosylation, etc., to regulate protein degradation or production, thereby affecting protein expression and activity [25–31].

We have previously demonstrated that SOX2OT is upregulated in pancreatic cancer tissues and is associated with poor prognosis in pancreatic cancer patients. In vitro study results have shown that overexpression of SOX2OT may lead to the malignant proliferation of pancreatic cancer cells. The close correlation between SOX2OT expression and pancreatic cancer was confirmed at tissue and cellular levels. We also revealed the in vivo binding of SOX2OT to the RNA binding protein FUS, which reduced the stability of FUS at the protein level, without changing the FUS mRNA expression [8, 9].

In this study, we performed tumor growth and metastasis experiments in nude mice and found that SOX2OT could significantly promote the growth and metastasis of pancreatic cancer cells in nude mice. Immunohistochemistry (Ki67) results also confirmed that SOX2OT expression was positively correlated with the proliferative activity of tumor cells. However, FUS had negative regulatory effects on the tumor growth and metastasis of pancreatic cancer cells and FUS overexpression could obviously reverse the carcinogenesis caused by SOX2OT. The present results revealed the effects of SOX2OT–FUS regulatory axis on tumorigenicity and metastasis of pancreatic cancer.

To determine whether the regulation of FUS protein by SOX2OT affects the migration and invasion of pancreatic cancer cells, we reversed the regulation of FUS protein by SOX2OT using ectopic expression of FUS. As a result, the promotion of migration and invasion of pancreatic cancer cell lines by SOX2OT were significantly attenuated through ectopic expression of FUS. These results suggested that SOX2OT may exert its effect of promotion of migration and invasion by affecting the stability of FUS protein.

Through RNA pull-down assay, we confirmed that SOX2OT could directly bind to FUS protein in vitro through its 5′ (1–887 nt) and 3′ (2663–3550 nt) regions. Whether the binding of SOX2OT to FUS affect the stability of protein (to achieve its regulatory function) was unknown. We treated all the groups of cells with CHX to block cell protein synthesis and determined FUS protein expression. The results showed that SOX2OT could promote the degradation of FUS protein. To clarify the mechanism underlying SOX2OT-mediated degradation of FUS, we treated pancreatic cancer cells with MG132 to block protein degradation via proteasome, and performed western blot analysis to determine the difference in FUS expression among all the groups of cells. The results suggested that the proteasome inhibitor MG132 could significantly attenuate the degradation of FUS protein by SOX2OT. We performed ubiquitination assay to confirm that SOX2OT could promote the ubiquitination of FUS. The above experiments demonstrated that SOX2OT may directly bind to FUS protein and promote its degradation via ubiquitination, thereby regulating its expression.

SOX2OT regulates the levels of a large number of mRNAs through modulating FUS in pancreatic cancer. GO analysis revealed that the SOX2OT–FUS regulated genes are mainly enriched for the regulators of gene expression that function at posttranscriptional and translational level. Meanwhile, many of these SOX2OT–FUS regulated genes have been found to directly involve in cancer cell apoptosis, such as PCDHGC3 [32], in cell proliferation, such as ZBED6 [33], in cell cycle, such as SCAMP5 [34] and ZNF84 [35], in cell migration and cell adhesion, such as SCAMP5 [34], and in tumor immunosuppression, such as ENTPD1-AS1 and IL10RB-DT [36, 37]. However, the specific molecular mechanisms of SOX2OT–FUS axis in pancreatic cancer remains to be further verified.

**Fig. 6:**
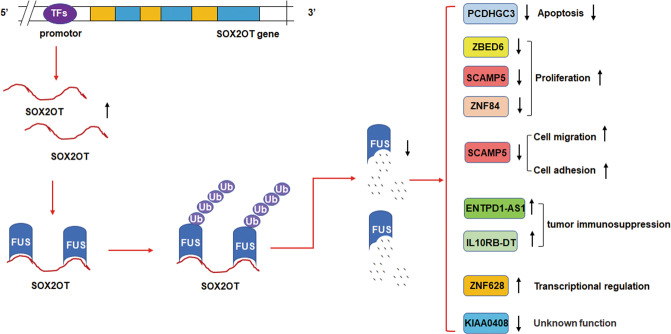
Schematic representation for the mechanism of SOX2OT–FUS axis in pancreatic cancer progression. SOX2OT may bind to FUS protein to promote its degradation via ubiquitination, thereby promoting cell proliferation, migration, and invasion, then affecting the development of pancreatic cancer.

In conclusion, SOX2OT may bind to FUS protein to promote its degradation via ubiquitination, thereby promoting cell proliferation, migration, and invasion, then affecting the development of pancreatic cancer (Fig. 6). The in-depth elaboration of the SOX2OT–FUS regulatory axis in pancreatic cancer may clarify the mechanism of action of SOX2OT and provide new ideas for pancreatic cancer treatment.

## Materials and methods

### Cell lines and cell culture

The stable SOX2OT overexpression PANC-1 cell line (PANC-1–SOX2OT), SOX2OT knockdown PANC-1 cell line (PANC-1–SOX2OT shRNA), and their control cell lines (PANC-1–Vector, PANC-1–Scramble shRNA) were prepared previously [9]. The cells were grown in Dulbecco’s modified Eagle’s medium (Wisent Inc., Montreal, QC, Canada) supplemented with 10% fetal calf serum (Wisent), 10 mM HEPES (Wisent), 2 mM L-glutamine (Wisent), 1 mM pyruvate sodium (Wisent), 100 units/ml penicillin (Wisent), and 100 μg/ml streptomycin (Wisent) at 37 °C in a humidified atmosphere containing 95% air and 5% CO_2_. To inhibit protein synthesis or degradation, cells were treated with either CHX (100 μg/ml, Sigma, St Louis, MO) for 0, 3, 6, and 12 h or MG132 (20 μM, Sigma) for 6 h along with DMSO vehicle controls.

### Ectopic expression of FUS

OBiO Biotechnology Co., Ltd (Shanghai, China) constructed the FUS overexpression lentiviruses and its control lentiviruses. The full-length human FUS was subcloned into pLenti-CMV-MCS-3FLAG H155 vector and verified by sequencing (Additional file 4). SOX2OT overexpression PANC-1 cells (PANC-1–SOX2OT) were infected with FUS overexpression lentiviruses or control lentiviruses. Stable cell line (PANC-1–SOX2OT–FUS) were selected by culturing the cells in media containing 5 μg/ml puromycin (Sigma). FUS expression was confirmed by western blot analysis.

### Quantitative RT-PCR

Total RNAs were prepared using the TRIzol reagent (Life Technologies, Carlsbad, CA, USA), according to the manufacturer’s protocol. After spectrophotometric quantification, 1 μg of total RNA was used for reverse transcription in a final volume of 20 μl with an iScript cDNA Synthesis Kit (Bio-Rad, Hercules, CA, USA) according to the manufacturer’s instructions. Quantitative PCR was performed using SYBR Green MasterMix (Life Technologies) in a StepOnePlus Real-time PCR System (Life Technologies). The relative gene expression was calculated by the subtraction of the Ct value of target genes and β-actin (control) gene by the 2^−ΔCT^ method [38]. The primer sequence of target genes and β-actin gene was shown in Additional file 5 (Table S4). Each quantitative PCR was performed in triplicate and independently repeated three times.

### Western blot analysis

Total cell lysates were collected as described previously [8]. Protein concentrations were measured by using the DC protein assay kit (Bio-Rad). Equal amounts of protein were electrophoresed using 12% sodium dodecyl sulfate polyacrylamide gel electrophoresis (SDS-PAGE) and transferred to a PVDF membrane (Bio-Rad). Nonspecific protein interactions were blocked by incubation in 5% nonfat dry milk in 0.1% Tween 20 (TBST) buffer at room temperature for 1 h and then washed with TBST. Membranes were then incubated at 4 °C overnight with primary antibodies in fresh blocking buffer. The antibody to FUS and β-Actin were from Santa Cruz Biotechnology (Dallas, Texas, USA). The blots were then washed and incubated with HRP-conjugated secondary antibodies (Beyotime, Shanghai, China) for 1 h at room temperature. The bands were visualized with Immobilon Western Chemilum HRP substrate (Merck Millipore, Darmstadt, Germany) using the FluorChem E System (ProteinSimple, Santa Clara, CA, USA). Prestained markers (Thermo Fisher Scientific) were used as internal molecular weight standards. Each blot was independently repeated three times.

### In vivo tumor growth and metastasis study

The animal studies were approved by the Institutional Animal Care and Use Committee of the First Affiliated Hospital of Nanjing Medical University, Nanjing, China. Five–six-week-old female nude mice (BALB/c-nude) were purchased from GemPharmatech Co., Ltd. (Nanjing, China). In total, three groups with six mice per group were used for the subcutaneous tumorigenesis models. PANC-1–Vector, PANC-1–SOX2OT, and PANC-1–SOX2OT–FUS cells (1 × 10^6^ cells/100 μl) were subcutaneously injected into the subaxillary fossa of right anterior limb in each mouse, separately. When a tumor was palpable, body weight and tumor volume were measured twice weekly. The tumor volume was calculated using the formula (width^2^ × length)/2. About 9 weeks (61 days) later, the mice were euthanized. After the mice were photographed, the subcutaneous tumors were taken, weighed, and photographed. Three groups with six mice per group were used for the tail vein metastasis model, PANC-1–Vector, PANC-1–SOX2OT and PANC-1–SOX2OT–FUS cells (5 × 10^6^ cells/100 μl) were separately injected into the tail vein of each mouse. Four weeks later, the mice were euthanized and lungs and livers were removed. Each tissue sample was cut in two, one was snap-frozen in liquid nitrogen until use and the other was and fixed in 4% paraformaldehyde (for immunohistochemistry).

### Immunohistochemistry

Immunohistochemistry was performed as follows. Four-micrometer-thick TMA sections were cut and mounted on poly-L-lysine-coated glass slides. Slides were deparaffinized in xylene, rehydrated in graded alcohol, and washed in tap water. Endogenous peroxidase was blocked by incubating the sections in 3% H_2_O_2_ for 5 min. Antigen retrieval was then performed for 20 min in 10 mmol/l sodium citrate buffer (pH 6.0) heated at 95 °C in a microwave oven, followed by a blocking step with 5% normal goat serum for 10 min at room temperature. After blocking, the sections were incubated at 4 °C overnight with anti-FUS antibody (Santa Cruz, 1:150 dilution) or Ki-67 antibody (Abcam, Cambridge, MA, USA, 1:150 dilution), followed by incubation with a secondary antibody (goat anti-rabbit/horseradish peroxidase, Santa Cruz, 1:200 dilution) at room temperature for 30 min. Finally, the sections were developed with diaminobenzidine substrate for about 5 min, and counterstained with hematoxylin.

### Cell wound healing assay

Cell wound healing assay was used to evaluate cell migration. Forty-eight hours after cell seeding, a monolayer was scraped with a 200 μl pipette tip to produce lesions of a constant length. After washing with PBS to remove the loose cells, phase images were taken by inversion fluorescence microscopy (DMi8, Leica) at 0, 24, 48, and 72 h, respectively. Image-Pro Plus software was used to measure the relative wound areas.

### Cellular transwell assay

Cell invasion was assessed using 24-well Millicell Hanging Cell Culture Inserts (PET membranes with 8 μm pores, Merck Millipore). The upper surface of each insert was coated with Matrigel (BD Biosciences Pharmingen), following the manufacturer’s protocol. Briefly, 10% (v/v) fetal bovine serum was used as the chemoattractant in the lower chamber. Overall, 5 × 10^4^ cells were seeded in the upper chamber and incubated at 37 °C for 48 h. Noninvading cells were removed from the upper surface of the membrane by wiping with cotton-tipped swabs. Cells on the lower surface of the membrane were stained with 0.1% crystal violet for 10 min and photographed by inversion fluorescence microscopy (DMi8, Leica). The cells in five random fields of view under ×200 magnification were counted. Finally, the stained cells were then soaked in 33% acetic acid and oscillated for 10 min. The absorbance of 33% acetic acid containing crystal violet was then assessed using a microplate reader (Tecan, Shanghai, China) at a 570 nm wavelength. The optical density (OD) value indirectly reflected the number of penetrated cells. OD values for triplicate membranes were reported as the mean ± SD and the experiments were repeated three times.

### RNA pull‑down assay

The RNA pull-down assay was performed using the Pierce™ magnetic RNA-protein pull-down kit (Thermo Fisher Scientific, MA, USA). Full-length and truncated fragments of SOX2OT were transcribed and biotin labeled in vitro from the pcDNA3.1(+) vector using the Ribo RNAmax-T7 Biotin Labeling Transcription Kit (C11002-1). The experimental procedures were carried out according to the manufacturer’s instructions. Briefly, 3 μg of biotin-labeled RNA was mixed with 2 mg of protein extract, and the mixture was incubated with Dynabeads MyOne Streptavidin T1 beads overnight at 4 °C. The RNA–protein complex was subjected to SDS-PAGE followed by western blot analysis.

### CHX‑chase assay

CHX-chase assay was performed using CHX (Sigma), an inhibitor of protein synthesis. The cells in each group were treated with 100 μg/ml of CHX and the expression of FUS protein was determined by western blot analysis at 0, 3, 6, and 12 h.

### Plasmid construction

For ubiquitination assay, Ub and FUS expression plasmids were constructed by OBiO Biotechnology Co. Ltd. HA and FLAG tag sequences were added in-frame to their N-terminal (HA-Ub and FLAG-FUS), respectively. All above cDNAs were cloned into pcDNA3.1(+) vector containing a puromycin resistance cassette and were under the control of CMV promoter. The sequences of these cDNAs were verified by Sanger sequencing.

### Ubiquitination assay

Cells were cotransfected with HA-Ub and FLAG-FUS plasmids using Lipofectamine 3000 (Promega, Madison, WI, USA), according to the manufacturer’s protocol. After 24 h of transfection, 20 μM of MG132 (Sigma) was added to the medium for 6 h, followed by cell lysis for IP. Cell lysate was prepared by briefly sonicating ten million cells in 1 ml ice precooling IP LYSIS/WASH buffer (0.025 M Tris [pH 7.4], 0.15 M NaCl, 0.001 M EDTA, 1% NP-40, 5% glycerol) supplemented with 1 × complete protease inhibitors [Roche]). The ubiquitination level of FLAG-FUS protein was detected by IP. IP was performed using Pierce classic IP kit (Thermo Fisher Scientific), according to the manufacturer’s protocol. The cell lysates were incubated with 5 μg anti-HA antibody (Beyotime) or normal rabbit IgG (Beyotime) overnight at 4 °C. The ubiquitinated proteins were retrieved, washed, and eluted in elution buffer and subjected to western blot using the anti-FLAG antibody (Beyotime) to detect ubiquitinated FLAG-FUS.

### RNA sequencing

RNA-seq of PANC-1–Vector, PANC-1–SOX2OT, and PANC-1–SOX2OT–FUS cells was carried out by OBiO Biotechnology Co., Ltd. Total RNA was isolated and purified using TRIzol reagent (Invitrogen, Carlsbad, CA, USA) following the manufacturer’s procedure. The RNA amount and purity of each sample was quantified using NanoDrop ND-1000 (NanoDrop, Wilmington, DE, USA). The RNA integrity was assessed by Bioanalyzer 2100 (Agilent, CA, USA) with RIN number > 7.0, and confirmed by electrophoresis with denaturing agarose gel. Poly(A) RNA was purified from 1 μg total RNA using Dynabeads Oligo (dT)25-61005 (Thermo Fisher, CA, USA) using two rounds of purification. Then, the poly(A) RNA was fragmented into small pieces using Magnesium RNA Fragmentation Module (NEB, cat.e6150, USA) under 94 °C for 5–7 min. Then, the cleaved RNA fragments were reverse-transcribed to create the cDNA by SuperScript™ II Reverse Transcriptase (Invitrogen, cat.1896649, USA), which were next used to synthesize U-labeled second-stranded DNAs with E. coli DNA polymerase I (NEB, cat.m0209, USA), RNase H (NEB, cat.m0297, USA), and dUTP Solution (Thermo Fisher, cat.R0133, USA). An A-base was then added to the blunt ends of each strand, preparing them for ligation to the indexed adapters. Each adapter contains a T-base overhang for ligating the adapter to the A-tailed fragmented DNA. Single- or dual-index adapters were ligated to the fragments, and size selection was performed with AMPure XP beads. After the heat-labile UDG enzyme (NEB, cat.m0280, USA) treatment of the U-labeled second-stranded DNAs, the ligated products were amplified with PCR by the following conditions: initial denaturation at 95 °C for 3 min; 8 cycles of denaturation at 98 °C for 15 s, annealing at 60 °C for 15 s, and extension at 72 °C for 30 s, and then final extension at 72 °C for 5 min. The average insert size for the final cDNA library was 300 ± 50 bp. At last, we performed the 2 × 150 bp paired-end sequencing (PE150) on an Illumina NovaSeq™ 6000 (LC-Bio Technologies Co., Ltd., Hangzhou, China) following the vendor’s recommended protocol.

Cutadapt software was used to remove the reads that contained adapter contamination. After removing the low quality bases and undetermined bases, we used HISAT2 software to map reads to the genome. The mapped reads of each sample were assembled using StringTie with default parameters. Then, all transcriptomes from all samples were merged to reconstruct a comprehensive transcriptome using gffcompare software. After the final transcriptome was generated, StringTie and Ballgown were used to estimate the expression levels of all transcripts and perform expression level for mRNAs by calculating FPKM (FPKM = [total_exon_fragments/mapped_reads (millions) × exon_length (kB)]). The differentially expressed mRNAs were selected with fold change > 2 or fold change < 0.5 and *p* value < 0.05 by R package edgeR or DESeq2, and then analysis GO enrichment and KEGG enrichment to the differentially expressed mRNAs. GO functional significance enrichment analysis first maps all significant differentially expressed genes to each term in the GO database, calculates the number of genes for each term, and then uses hypergeometric test to find out the GO entries significantly enriched in significant differentially expressed genes compared with the whole-genome background. The significant enrichment analysis of pathway takes KEGG pathway as the unit, and uses hypergeometric test to find out the pathways significantly enriched in the significantly differentially expressed genes compared with the whole-genome background.

### Statistical analysis

Statistical analysis was performed using the SPSS software (Version 15.0). Quantitative data were presented as mean ± SD. Differences in the mean of two samples were analyzed by Student’s *t* test. In addition, owing to the small number of mice used, the data obtained using the tumor models were analyzed by Fisher’s exact test. All statistical tests were two-tailed exact tests with a *p* < 0.05 considered significant.

## Supplementary information

Table S1

Table S2

Table S3

Additional file 4

Table S4

## Data Availability

The datasets used and/or analyzed during the current study are available from the corresponding author on reasonable request.

## References

[1] Siegel RL, Miller KD, Jemal A (2020). Cancer statistics, 2020. CA Cancer J Clin.

[2] Chen W, Zheng R, Baade PD, Zhang S, Zeng H, Bray F (2016). Cancer statistics in China, 2015. CA Cancer J Clin.

[3] Adams BD, Parsons C, Walker L, Zhang WC, Slack FJ (2017). Targeting noncoding RNAs in disease. J Clin Investig.

[4] Kung JT, Colognori D, Lee JT (2013). Long noncoding RNAs: past, present, and future. Genetics.

[5] Zheng S, Chen H, Wang Y, Gao W, Fu Z, Zhou Q (2016). Long non-coding RNA LOC389641 promotes progression of pancreatic ductal adenocarcinoma and increases cell invasion by regulating E-cadherin in a TNFRSF10A-related manner. Cancer Lett.

[6] Wang S, Liang K, Hu Q, Li P, Song J, Yang Y (2017). JAK2-binding long noncoding RNA promotes breast cancer brain metastasis. J Clin Investig.

[7] Zhang E, He X, Zhang C, Su J, Lu X, Si X (2018). A novel long noncoding RNA HOXC-AS3 mediates tumorigenesis of gastric cancer by binding to YBX1. Genome Biol.

[8] Zhang JJ, Zhu Y, Zhang XF, Liu DF, Wang Y, Yang C (2017). Yin Yang-1 suppresses pancreatic ductal adenocarcinoma cell proliferation and tumor growth by regulating SOX2OT-SOX2 axis. Cancer Lett.

[9] Chen L, Zhang J, Chen Q, Ge W, Meng L, Huang X (2020). Long noncoding RNA SOX2OT promotes the proliferation of pancreatic cancer by binding to FUS. Int J Cancer.

[10] Lagier-Tourenne C, Polymenidou M, Cleveland DW (2010). TDP-43 and FUS/TLS: emerging roles in RNA processing and neurodegeneration. Hum Mol Genet..

[11] Harrison AF, Shorter J (2017). RNA-binding proteins with prion-like domains in health and disease. Biochem J.

[12] Sato S, Idogawa M, Honda K, Fujii G, Kawashima H, Takekuma K (2015). Beta-catenin interacts with the FUS proto-oncogene product and regulates pre-mRNA splicing. Gastroenterology.

[13] Brooke GN, Culley RL, Dart DA, Mann DJ, Gaughan L, McCracken SR (2011). FUS/TLS is a novel mediator of androgen-dependent cell-cycle progression and prostate cancer growth. Cancer Res.

[14] Ke H, Zhao L, Feng X, Xu H, Zou L, Yang Q (2016). NEAT1 is required for survival of breast Cancer cells through FUS and miR-548. Gene Regul Syst Biol.

[15] Zhu H, Zheng T, Yu J, Zhou L, Wang L (2018). LncRNA XIST accelerates cervical cancer progression via upregulating Fus through competitively binding with miR-200a. Biomed Pharmacother.

[16] Bao L, Yuan L, Li P, Bu Q, Guo A, Zhang H (2018). FUS-LATS1/2 axis inhibits hepatocellular carcinoma progression via activating hippopathway. Cell Physiol Biochem.

[17] Su R, Cao S, Ma J, Liu Y, Liu X, Zheng J (2017). Knockdown of SOX2OT inhibits the malignant biological behaviors of glioblastoma stem cells via up-regulating the expression of miR-194-5p and miR-122. Mol Cancer.

[18] Shahryari A, Rafiee MR, Fouani Y, Oliae NA, Samaei NM, Shafiee M (2014). Two novel splice variants of SOX2OT, SOX2OT-S1, and SOX2OT-S2 are coupregulated with SOX2 and OCT4 in esophageal squamous cell carcinoma. Stem Cells.

[19] Wang W, Chen D, Zhu K (2018). SOX2OT variant 7 contributes to the synergistic interaction between EGCG and doxorubicin to kill osteosarcoma via autophagy and stemness inhibition. J Exp Clin Cancer Res.

[20] Li Z, Jiang P, Li J, Peng M, Zhao X, Zhang X (2018). Tumor-derived exosomal lnc-Sox2ot promotes EMT and stemness by acting as a ceRNA in pancreatic ductal adenocarcinoma. Oncogene.

[21] Wang KC, Chang HY (2011). Molecular mechanisms of long noncoding RNAs. Mol Cell.

[22] Li X, Wu Z, Fu X, Han W (2014). lncRNAs: insights into their function and mechanics in underlying disorders. Mutat Res Rev Mutat Res.

[23] Gandhi M, Caudron-Herger M, Diederichs S (2018). RNA motifs and combinatorial prediction of interactions, stability and localization of noncoding RNAs. Nat Struct Mol Biol.

[24] Mishra K, Kanduri C (2019). Understanding long noncoding RNA and chromatin interactions: what we know so far. Noncoding RNA.

[25] Lan Y, Xiao X, He Z, Luo Y, Wu C, Li L (2018). Long noncoding RNA OCC-1 suppresses cell growth through destabilizing HuR protein in colorectal cancer. Nucleic Acids Res.

[26] Liu B, Sun L, Liu Q, Gong C, Yao Y, Lv X (2015). A cytoplasmic NF-κB interacting long noncoding RNA blocks IκB phosphorylation and suppresses breast cancer metastasis. Cancer Cell.

[27] Taniue K, Kurimoto A, Sugimasa H, Nasu E, Takeda Y, Iwasaki K (2016). Long noncoding RNA UPAT promotes colon tumorigenesis by inhibiting degradation of UHRF1. Proc Natl Acad Sci USA.

[28] Liang Y, Chen X, Wu Y, Li J, Zhang S, Wang K (2018). LncRNA CASC9 promotes esophageal squamous cell carcinoma metastasis through upregulating LAMC2 expression by interacting with the CREB-binding protein. Cell Death Differ.

[29] Ni W, Zhang Y, Zhan Z, Ye F, Liang Y, Huang J (2017). A novel lncRNA uc.134 represses hepatocellular carcinoma progression by inhibiting CUL4A-mediated ubiquitination of LATS1. J Hematol Oncol.

[30] Tang J, Yan T, Bao Y, Shen C, Yu C, Zhu X (2019). LncRNA GLCC1 promotes colorectal carcinogenesis and glucose metabolism by stabilizing c-Myc. Nat Commun..

[31] Wang Y, Lu JH, Wu QN, Jin Y, Wang DS, Chen YX (2019). LncRNA LINRIS stabilizes IGF2BP2 and promotes the aerobic glycolysis in colorectal cancer. Mol Cancer.

[32] Dallosso AR, Øster B, Greenhough A, Thorsen K, Curry TJ, Owen C (2012). Long-range epigenetic silencing of chromosome 5q31 protocadherins is involved in early and late stages of colorectal tumorigenesis through modulation of oncogenic pathways. Oncogene.

[33] Akhtar Ali M, Younis S, Wallerman O, Gupta R, Andersson L, Sjöblom T (2015). Transcriptional modulator ZBED6 affects cell cycle and growth of human colorectal cancer cells. Proc Natl Acad Sci USA.

[34] Mao F, Duan H, Allamyradov A, Xin Z, Du Y, Wang X (2021). Expression and prognostic analyses of SCAMPs in pancreatic adenocarcinoma. Aging.

[35] Strzeszewska-Potyrała A, Staniak K, Czarnecka-Herok J, Rafiee MR, Herok M, Mosieniak G (2021). Chromatin-directed proteomics identifies ZNF84 as a p53-independent regulator of p21 in genotoxic stress response. Cancers.

[36] Zhou M, Zhang Z, Zhao H, Bao S, Cheng L, Sun J (2018). An immune-related six-lncRNA signature to improve prognosis prediction of glioblastoma multiforme. Mol Neurobiol.

[37] Jiang Y, Gou X, Wei Z, Tan J, Yu H, Zhou X (2020). Bioinformatics profling integrating a three immune-related long non-coding RNA signature as a prognostic model for clear cell renal cell carcinoma. Cancer Cell Int.

[38] Livak KJ, Schmittgen TD (2001). Analysis of relative gene expression data using realtime quantitative PCR and the 2^−ΔΔCT^ method. Methods.

