# Supplementary material for: Long noncoding RNA SOX2OT promotes pancreatic cancer cell migration and invasion through destabilizing FUS protein via ubiquitination
Source: Cell Death Discov. 2021 Sep 22;7:261. doi: 10.1038/s41420-021-00640-8 (PMC8458496; doi:10.1038/s41420-021-00640-8)
Supplement: Supplementary file 4 — Additional file 4 [file 41420_2021_640_MOESM4_ESM.doc]

CGTGTACGGTGGGAGGTCTATATAAGCAGAGCTCGTTTAGTGAACCGTCAGATCGAATTCGCCACCATGGCCTCAAACGATTATACCCAACAAGCAACCCAAAGCTATGGGGCCTACCCCACCCAGCCCGGGCAGGGCTATTCCCAGCAGAGCAGTCAGCCCTACGGACAGCAGAGTTACAGTGGTTATAGCCAGTCCACGGACACTTCAGGCTATGGCCAGAGCAGCTATTCTTCTTATGGCCAGAGCCAGAACACAGGCTATGGAACTCAGTCAACTCCCCAGGGATATGGCTCGACTGGCGGCTATGGCAGTAGCCAGAGCTCCCAATCGTCTTACGGGCAGCAGTCCTCCTACCCTGGCTATGGCCAGCAGCCAGCTCCCAGCAGCACCTCGGGAAGTTACGGTAGCAGTTCTCAGAGCAGCAGCTATGGGCAGCCCCAGAGTGGGAGCTACAGCCAGCAGCCTAGCTATGGTGGACAGCAGCAAAGCTATGGACAGCAGCAAAGCTATAATCCCCCTCAGGGCTATGGACAGCAGAACCAGTACAACAGCAGCAGTGGTGGTGGAGGTGGAGGTGGAGGTGGAGGTAACTATGGCCAAGATCAATCCTCCATGAGTAGTGGTGGTGGCAGTGGTGGCGGTTATGGCAATCAAGACCAGAGTGGTGGAGGTGGCAGCGGTGGCTATGGACAGCAGGACCGTGGAGGCCGCGGCAGGGGTGGCAGTGGTGGCGGCGGCGGCGGCGGCGGTGGTGGTTACAACCGCAGCAGTGGTGGCTATGAACCCAGAGGTCGTGGAGGTGGCCGTGGAGGCAGAGGTGGCATGGGCGGAAGTGACCGTGGTGGCTTCAATAAATTTGGTGGCCCTCGGGACCAAGGATCACGTCATGACTCCGAACAGGATAATTCAGACAACAACACCATCTTTGTGCAAGGCCTGGGTGAGAATGTTACAATTGAGTCTGTGGCTGATTACTTCAAGCAGATTGGTATTATTAAGACAAACAAGAAAACGGGACAGCCCATGATTAATTTGTACACAGACAGGGAAACTGGCAAGCTGAAGGGAGAGGCAACGGTCTCTTTTGATGACCCACCTTCAGCTAAAGCAGCTATTGACTGGTTTGATGGTAAAGAATTCTCCGGAAATCCTATCAAGGTCTCATTTGCTACTCGCCGGGCAGACTTTAATCGGGGTGGTGGCAATGGTCGTGGAGGCCGAGGGCGAGGAGGACCCATGGGCCGTGGAGGCTATGGAGGTGGTGGCAGTGGTGGTGGTGGCCGAGGAGGATTTCCCAGTGGAGGTGGTGGCGGTGGAGGACAGCAGCGAGCTGGTGACTGGAAGTGTCCTAATCCCACCTGTGAGAATATGAACTTCTCTTGGAGGAATGAATGCAACCAGTGTAAGGCCCCTAAACCAGATGGCCCAGGAGGGGGACCAGGTGGCTCTCACATGGGGGGTAACTACGGGGATGATCGTCGTGGTGGCAGAGGAGGCTATGATCGAGGCGGCTACCGGGGCCGCGGCGGGGACCGTGGAGGCTTCCGAGGGGGCCGGGGTGGTGGGGACAGAGGTGGCTTTGGCCCTGGCAAGATGGATTCCAGGGGTGAGCACAGACAGGATCGCAGGGAGAGGCCGTATGAATTCGACTACAAGGATGACGATGACAAGGATTACAAAGACGACGATGATAAGGACTATAAGGATGATGACGACAA

1 50

SEQ (1) CGTGTACGGTGGGAGGTCTATATAAGCAGAGCTCGTTTAGTGAACCGTCA

FUS (1) --------------------------------------------------

51 100

SEQ (51) GATCGAATTCGCCACCATGGCCTCAAACGATTATACCCAACAAGCAACCC

FUS (1) ----------------ATGGCCTCAAACGATTATACCCAACAAGCAACCC

101 150

SEQ (101) AAAGCTATGGGGCCTACCCCACCCAGCCCGGGCAGGGCTATTCCCAGCAG

FUS (35) AAAGCTATGGGGCCTACCCCACCCAGCCCGGGCAGGGCTATTCCCAGCAG

151 200

SEQ (151) AGCAGTCAGCCCTACGGACAGCAGAGTTACAGTGGTTATAGCCAGTCCAC

FUS (85) AGCAGTCAGCCCTACGGACAGCAGAGTTACAGTGGTTATAGCCAGTCCAC

201 250

SEQ (201) GGACACTTCAGGCTATGGCCAGAGCAGCTATTCTTCTTATGGCCAGAGCC

FUS (135) GGACACTTCAGGCTATGGCCAGAGCAGCTATTCTTCTTATGGCCAGAGCC

251 300

SEQ (251) AGAACACAGGCTATGGAACTCAGTCAACTCCCCAGGGATATGGCTCGACT

FUS (185) AGAACACAGGCTATGGAACTCAGTCAACTCCCCAGGGATATGGCTCGACT

301 350

SEQ (301) GGCGGCTATGGCAGTAGCCAGAGCTCCCAATCGTCTTACGGGCAGCAGTC

FUS (235) GGCGGCTATGGCAGTAGCCAGAGCTCCCAATCGTCTTACGGGCAGCAGTC

351 400

SEQ (351) CTCCTACCCTGGCTATGGCCAGCAGCCAGCTCCCAGCAGCACCTCGGGAA

FUS (285) CTCCTACCCTGGCTATGGCCAGCAGCCAGCTCCCAGCAGCACCTCGGGAA

401 450

SEQ (401) GTTACGGTAGCAGTTCTCAGAGCAGCAGCTATGGGCAGCCCCAGAGTGGG

FUS (335) GTTACGGTAGCAGTTCTCAGAGCAGCAGCTATGGGCAGCCCCAGAGTGGG

451 500

SEQ (451) AGCTACAGCCAGCAGCCTAGCTATGGTGGACAGCAGCAAAGCTATGGACA

FUS (385) AGCTACAGCCAGCAGCCTAGCTATGGTGGACAGCAGCAAAGCTATGGACA

501 550

SEQ (501) GCAGCAAAGCTATAATCCCCCTCAGGGCTATGGACAGCAGAACCAGTACA

FUS (435) GCAGCAAAGCTATAATCCCCCTCAGGGCTATGGACAGCAGAACCAGTACA

551 600

SEQ (551) ACAGCAGCAGTGGTGGTGGAGGTGGAGGTGGAGGTGGAGGTAACTATGGC

FUS (485) ACAGCAGCAGTGGTGGTGGAGGTGGAGGTGGAGGTGGAGGTAACTATGGC

601 650

SEQ (601) CAAGATCAATCCTCCATGAGTAGTGGTGGTGGCAGTGGTGGCGGTTATGG

FUS (535) CAAGATCAATCCTCCATGAGTAGTGGTGGTGGCAGTGGTGGCGGTTATGG

651 700

SEQ (651) CAATCAAGACCAGAGTGGTGGAGGTGGCAGCGGTGGCTATGGACAGCAGG

FUS (585) CAATCAAGACCAGAGTGGTGGAGGTGGCAGCGGTGGCTATGGACAGCAGG

701 750

SEQ (701) ACCGTGGAGGCCGCGGCAGGGGTGGCAGTGGTGGCGGCGGCGGCGGCGGC

FUS (635) ACCGTGGAGGCCGCGGCAGGGGTGGCAGTGGTGGCGGCGGCGGCGGCGGC

751 800

SEQ (751) GGTGGTGGTTACAACCGCAGCAGTGGTGGCTATGAACCCAGAGGTCGTGG

FUS (685) GGTGGTGGTTACAACCGCAGCAGTGGTGGCTATGAACCCAGAGGTCGTGG

801 850

SEQ (801) AGGTGGCCGTGGAGGCAGAGGTGGCATGGGCGGAAGTGACCGTGGTGGCT

FUS (735) AGGTGGCCGTGGAGGCAGAGGTGGCATGGGCGGAAGTGACCGTGGTGGCT

851 900

SEQ (851) TCAATAAATTTGGTGGCCCTCGGGACCAAGGATCACGTCATGACTCCGAA

FUS (785) TCAATAAATTTGGTGGCCCTCGGGACCAAGGATCACGTCATGACTCCGAA

901 950

SEQ (901) CAGGATAATTCAGACAACAACACCATCTTTGTGCAAGGCCTGGGTGAGAA

FUS (835) CAGGATAATTCAGACAACAACACCATCTTTGTGCAAGGCCTGGGTGAGAA

951 1000

SEQ (951) TGTTACAATTGAGTCTGTGGCTGATTACTTCAAGCAGATTGGTATTATTA

FUS (885) TGTTACAATTGAGTCTGTGGCTGATTACTTCAAGCAGATTGGTATTATTA

1001 1050

SEQ (1001) AGACAAACAAGAAAACGGGACAGCCCATGATTAATTTGTACACAGACAGG

FUS (935) AGACAAACAAGAAAACGGGACAGCCCATGATTAATTTGTACACAGACAGG

1051 1100

SEQ (1051) GAAACTGGCAAGCTGAAGGGAGAGGCAACGGTCTCTTTTGATGACCCACC

FUS (985) GAAACTGGCAAGCTGAAGGGAGAGGCAACGGTCTCTTTTGATGACCCACC

1101 1150

SEQ (1101) TTCAGCTAAAGCAGCTATTGACTGGTTTGATGGTAAAGAATTCTCCGGAA

FUS (1035) TTCAGCTAAAGCAGCTATTGACTGGTTTGATGGTAAAGAATTCTCCGGAA

1151 1200

SEQ (1151) ATCCTATCAAGGTCTCATTTGCTACTCGCCGGGCAGACTTTAATCGGGGT

FUS (1085) ATCCTATCAAGGTCTCATTTGCTACTCGCCGGGCAGACTTTAATCGGGGT

1201 1250

SEQ (1201) GGTGGCAATGGTCGTGGAGGCCGAGGGCGAGGAGGACCCATGGGCCGTGG

FUS (1135) GGTGGCAATGGTCGTGGAGGCCGAGGGCGAGGAGGACCCATGGGCCGTGG

1251 1300

SEQ (1251) AGGCTATGGAGGTGGTGGCAGTGGTGGTGGTGGCCGAGGAGGATTTCCCA

FUS (1185) AGGCTATGGAGGTGGTGGCAGTGGTGGTGGTGGCCGAGGAGGATTTCCCA

1301 1350

SEQ (1301) GTGGAGGTGGTGGCGGTGGAGGACAGCAGCGAGCTGGTGACTGGAAGTGT

FUS (1235) GTGGAGGTGGTGGCGGTGGAGGACAGCAGCGAGCTGGTGACTGGAAGTGT

1351 1400

SEQ (1351) CCTAATCCCACCTGTGAGAATATGAACTTCTCTTGGAGGAATGAATGCAA

FUS (1285) CCTAATCCCACCTGTGAGAATATGAACTTCTCTTGGAGGAATGAATGCAA

1401 1450

SEQ (1401) CCAGTGTAAGGCCCCTAAACCAGATGGCCCAGGAGGGGGACCAGGTGGCT

FUS (1335) CCAGTGTAAGGCCCCTAAACCAGATGGCCCAGGAGGGGGACCAGGTGGCT

1451 1500

SEQ (1451) CTCACATGGGGGGTAACTACGGGGATGATCGTCGTGGTGGCAGAGGAGGC

FUS (1385) CTCACATGGGGGGTAACTACGGGGATGATCGTCGTGGTGGCAGAGGAGGC

1501 1550

SEQ (1501) TATGATCGAGGCGGCTACCGGGGCCGCGGCGGGGACCGTGGAGGCTTCCG

FUS (1435) TATGATCGAGGCGGCTACCGGGGCCGCGGCGGGGACCGTGGAGGCTTCCG

1551 1600

SEQ (1551) AGGGGGCCGGGGTGGTGGGGACAGAGGTGGCTTTGGCCCTGGCAAGATGG

FUS (1485) AGGGGGCCGGGGTGGTGGGGACAGAGGTGGCTTTGGCCCTGGCAAGATGG

1601 1650

SEQ (1601) ATTCCAGGGGTGAGCACAGACAGGATCGCAGGGAGAGGCCGTATGAATTC

FUS (1535) ATTCCAGGGGTGAGCACAGACAGGATCGCAGGGAGAGGCCGTAT------

1651 1700

SEQ (1651) GACTACAAGGATGACGATGACAAGGATTACAAAGACGACGATGATAAGGA

FUS (1579) --------------------------------------------------

1701 1721

SEQ (1701) CTATAAGGATGATGACGACAA

FUS (1579) ---------------------
