# Supplementary material for: Long noncoding RNA SOX2OT promotes pancreatic cancer cell migration and invasion through destabilizing FUS protein via ubiquitination
Source: Cell Death Discov. 2021 Sep 22;7:261. doi: 10.1038/s41420-021-00640-8 (PMC8458496; doi:10.1038/s41420-021-00640-8)
Supplement: Supplementary file 5 — Table S4 [file 41420_2021_640_MOESM5_ESM.docx]

**Table S1. qPCR Primers used for detecting mRNAs expression**

| Genes | Primer sequence(5’-3’) |
| --- | --- |
| ZNF628 | Forward:5’-GACCAGCCCCAGAAGTAACC-3’  Reverse:5’-ACTGGAATTGGAGAGCTGGC-3’ |
| MT-RNR2 | Forward:5’-ACCGGAGTAATCCAGGTCGG-3’  Reverse:5’-AGGCGCTTTGTGAAGTAGGC-3’ |
| CARHSP1 | Forward:5’-CCTGCACATCTCTGATGTGGA-3’  Reverse:5’-TGGTGCCAGGTGAGTGATGA-3’ |
| KRT8 | Forward:5’-CGAGGATATTGCCAACCGCAG-3’  Reverse:5’-CCTCAATCTCAGCCTGGAGCC-3’ |
| KLHL13 | Forward:5’-TGCAGCCAGTTATGCAGTCA-3’  Reverse:5’- TTACTCTGTCCGCCAACCAC-3’ |
| MMD | Forward:5’-CTGTCTGATGACTGCTGG-3’  Reverse:5’-GCATAAGAAGCAGCAATG-3’ |
| AHRR | Forward:5’-CTTAATGGCTTTGCTCTGGTCG-3’  Reverse:5’-TGCATTACATCCGTCTGATGGA-3’ |
| KIAA0408 | Forward:5’-TAGCAGAGAGCAGTAGCCCA-3’  Reverse:5’-GGGCTTGAGATCCTCCGATG-3’ |
| β-actin | Forward:5’-AGCGAGCATCCCCCAAAGTT-3’  Reverse:5’-GGGCACGAAGGCTCATCATT-3’ |
